# Deep Brain Stimulation Modified Autism-Like Deficits via the Serotonin System in a Valproic Acid-Induced Rat Model

**DOI:** 10.3390/ijms19092840

**Published:** 2018-09-19

**Authors:** Han-Fang Wu, Yi-Ju Chen, Ming-Chia Chu, Ya-Ting Hsu, Ting-Yi Lu, I-Tuan Chen, Po See Chen, Hui-Ching Lin

**Affiliations:** 1 Department and Institute of Physiology, School of Medicine, National Yang-Ming University, Taipei 11221, Taiwan; sindy7941@gmail.com (H.-F.W.); m0935540692@gmail.com (Y.-J.C.); sanwy47507@gmail.com (M.-C.C.); happylullaby@hotmail.com (Y.-T.H.); b0133021@gmail.com (T.-Y.L.); tim8315@gmail.com (I.-T.C.); 2 Department of Psychiatry, National Cheng Kung University Hospital, College of Medicine, National Cheng Kung University, Tainan 70101, Taiwan; chenps@mail.ncku.edu.tw; 3 Addiction Research Center, National Cheng Kung University, Tainan 70101, Taiwan; 4 Brain Research Center, National Yang-Ming University, Taipei 11221, Taiwan; 5 Ph.D. Program for Neural Regenerative Medicine, College of Medical Science and Technology, Taipei Medical University, Taipei 11031, Taiwan

**Keywords:** deep brain stimulation, autism spectrum disorder, valproic acid, serotonin system

## Abstract

Deep brain stimulation (DBS) is known to be a promising treatment for resistant depression, which acts via the serotonin (5-hydroxytryptamine, 5-HT) system in the infralimbic prefrontal cortex (ILPFC). Previous study revealed that dysfunction of brain 5-HT homeostasis is related to a valproate (VPA)-induced rat autism spectrum disorder (ASD) model. Whether ILPFC DBS rescues deficits in VPA-induced offspring through the 5-HT system is not known. Using VPA-induced offspring, we therefore explored the effect of DBS in autistic phenotypes and further investigated the underlying mechanism. Using combined behavioral and molecular approaches, we observed that applying DBS and 5-HT_1A_ receptor agonist treatment with 8-hydroxy-2-(di-n-propylamino)tetralin (8-OH-DPAT) reversed sociability deficits, anxiety and hyperactivity in the VPA-exposed offspring. We then administered the selective 5-HT_1A_ receptor antagonist *N*-[2-[4-(2-Methoxyphenyl)-1-piperazinyl]ethyl]-*N*-2-pyridinylcyclohexanecarboxamide maleate (WAY 100635), following which the effect of DBS in terms of improving autistic behaviors was blocked in the VPA-exposed offspring. Furthermore, we found that both 8-OH-DPAT and DBS treatment rescued autistic behaviors by decreasing the expressions of NR2B subunit of *N*-methyl-*D*-aspartate receptors (NMDARs) and the β_3_ subunit of γ-aminobutyric acid type A receptors (GABA_A_R) in the PFC region. These results provided the first evidence of characteristic behavioral changes in VPA-induced offspring caused by DBS via the 5-HT system in the ILPFC.

## 1. Introduction

Autism spectrum disorder (ASD) is a neurodevelopmental disorder characterized by impairment of social interaction, and repetitive and restricted-interests behaviors [1,2]. ASD is considered to be caused by an imbalance between excitation and inhibition (E/I) in neural circuits, resulting in impairment of social and emotional systems [3]. However, there is no effective medication to address the core characteristic symptoms of ASD in the clinical setting. Deep brain stimulation (DBS) has been demonstrated to control the motor symptoms of Parkinson’s disease (PD); however, it has also emerged as a therapy for psychiatric diseases, including obsessive-compulsive disorder (OCD), depression, Alzheimer’s disease and drug addiction, in recent years [4,5,6,7,8,9]. Clinical evidence suggests that DBS modulates dysfunctional limbic networks and their effects on neuropsychiatric disorders, including OCD, treatment-resistant depression (TRD), and Tourette’s syndrome [10]. Furthermore, DBS for severe ASD has been used successfully in some autistic patients [11,12,13]. DBS has been proposed to inhibit or excite local neuron activity depending on the neuronal composition of the stimulated nuclei [14]. As DBS modulates the E/I balance, it is a potential therapy for ASD.

Patients with ASD are at increased risk of comorbid symptoms of OCD and anxiety disorders [15]. The success of DBS for the treatment of OCD and anxiety, targeting at the nucleus accumbens (NAc) and the ventral internal capsule/ventral striatum (VC/VS), respectively, has been proven [16,17]. A previous study reported that in OCD patients with accompanying serotonergic deficits, NAc DBS increased the release of serotonin in brain areas of the PFC in rats, which suggested that DBS may modulate serotonin to improve OCD in patients with ASD [18]. DBS of the NAc has also been considered a target for treatment in patients with major depressive disorder [19]. Stimulation of the NAc enhances the medial prefrontal cortex (mPFC) serotonin (5-hydroxytryptamine, 5-HT) level and further alters the mPFC neuronal activity through activation of corticostriatal fibers [18,20]. Veerakumar et al. demonstrated that PFC DBS restored the dorsal raphe nucleus (DRN) intrinsic excitability and inhibitory input, which receives 5-HT projection from the PFC, promoting an antidepressant response [21]. Furthermore, the behavioral effect of DBS was blocked by 5-HT depletion, suggesting that DBS is involved in regulating the 5-HT system for an antidepressant effect [22,23]. DBS normalized the γ-aminobutyric acid (GABA) deficit and further elevated the functional connectivity in patients with depression [24,25]. Previous studies have demonstrated that systemic depletion of 5-HT increased the excitatory postsynaptic potentials, suggesting that upregulation of the glutamatergic system mediates the decreased 5-HT level, which may further result in abnormal behaviors [26,27]. Increasing evidence has demonstrated that activation of 5-HT_1_, 5HT_2_ and 5-HT_3_ receptors is involved in inhibiting GABAergic in PFC pyramidal neurons [28]. Despite promising results for the treatment of depression, the therapeutic effects of PFC DBS in ASD remain largely unknown.

More than 25% of autistic children exhibit an elevated blood 5-HT level or hyperserotonemia, which was the first biomarker of ASD to be identified [29]. Alteration of the brain 5-HT synthesis capacity is decreased in autistic children suggesting that dysfunction of the serotonin system is related to the pathogenesis of ASD [30,31,32,33]. Further evidence revealed that mice lacking brain serotonin by null mutation in the gene for tryptophan hydroxylase 2 (TPH2) showed deficits in social interaction and displayed highly repetitive behaviors [34]. Autistic children demonstrate a reduced serotonin transporter (SERT) binding capacity in the mPFC [35]. Previous studies revealed altered activity of the mPFC during tasks that required a conscious social-emotional response in an ASD group [36,37]. Abnormal serotonergic neuronal migration has been reported upon prenatal valproic acid (VPA) exposure [38,39]. In a VPA-induced ASD model, SERT was increased to improve autistic phenotypes by treatment with a 5-HT_1A_ receptor agonist [40]. Previous studies have indicated that ASD is accompanied by abnormality of the 5-HT system, and DBS restores the 5-HT system, further exerting an antidepressant effect [21,29]. We hypothesized that DBS could improve autistic symptoms via modulation of the 5-HT system in VPA-exposed offspring.

We built upon ILPFC DBS performed in an existing VPA-induced ASD model, which mimics the environmental toxins released by plasticizers [41]. In the current study, we examined whether ILPFC DBS and a 5-HT_1A_ receptor agonist reversed the decreased social interaction, increased anxiety and increased locomotion in a VPA-induced ASD model. We further examined whether DBS restored glutamatergic or GABAergic neurons via modulation of the 5-HT system in VPA-exposed offspring.

## 2. Results

### 2.1. DBS Rescued Behavioral Abnormalities in VPA-Exposed Offspring

**Figure 1:**
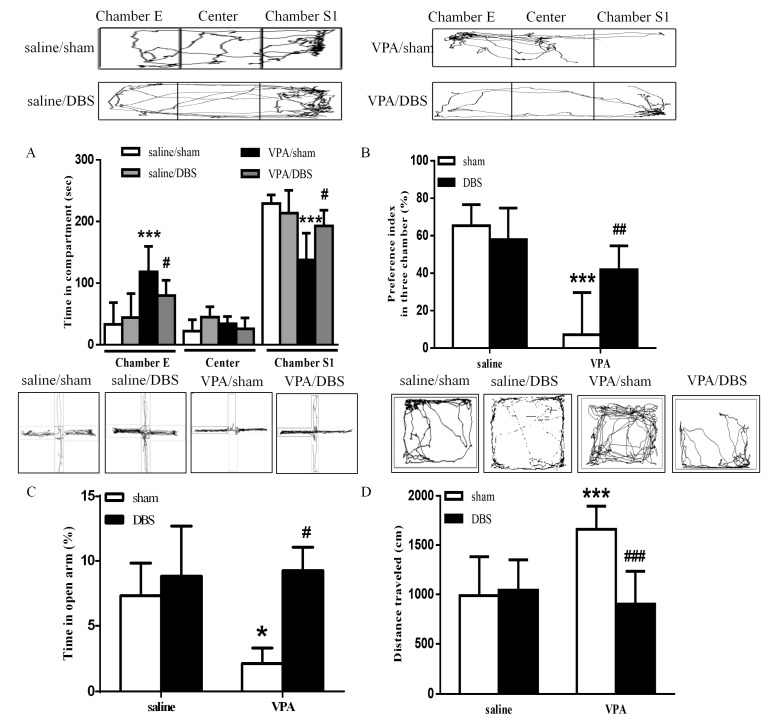
ILPFC DBS improved social interaction, anxiety and hyperlocomotion in the VPA-exposed offspring. (**A**) Time spent by the VPA-exposed offspring in chamber E, the center chamber, and chamber S1 during the three-chamber social interaction test following seven days of DBS treatment; (**B**) preference indices of the saline- and VPA-exposed offspring after DBS treatment; (**C**) bar chart showing the percentage of time spent in the open arms after DBS treatment; (**D**) bar chart revealing the total distance traveled in the open field test. * *p* < 0.05 vs. saline/sham; *** *p* < 0.001 vs. saline/sham; ^#^ *p* < 0.05 vs. VPA/sham; ^##^ *p* < 0.01 vs. VPA/sham; ^###^ *p* < 0.001 vs. VPA/sham. Sample sizes (*n*): saline/sham *n* = 7, saline/DBS *n* = 5, VPA/sham *n* = 9, VPA/DBS *n* = 7.

The social interaction test was conducted after seven days of chronic ILPFC DBS. The results revealed that ILPFC DBS resulted in a dramatic increase in the time spent in chamber S1 and decreased the time spent in chamber E in the VPA-exposed offspring (Figure 1A; saline/sham *n* = 7, saline/DBS *n* = 5, VPA/sham *n* = 9, VPA/DBS *n* = 7; two-way ANOVA revealed the time spent in chamber S1, pre-DBS *F*_(1, 24)_ = 24.67, *p* < 0.001; post-DBS *F*_(1, 24)_ = 4.304, *p* < 0.05; interaction *F*_(1, 24)_ = 5.348, *p* < 0.05; two-way ANOVA revealed the time spent in chamber E, pre-DBS *F*_(1, 24)_ = 24.84, *p* < 0.001; post-DBS *F*_(1, 24)_ = 4.319, *p* < 0.05; interaction *F*_(1, 24)_ = 4.443, *p* < 0.05). The decreased social preference index was reversed by ILPFC DBS in the VPA-exposed offspring (Figure 1B; saline/sham *n* = 7, saline/DBS *n* = 5, VPA/sham *n* = 9, VPA/DBS *n* = 7; two-way ANOVA, pre-DBS *F*_(1, 24)_ = 31.95, *p* < 0.001; post-DBS *F*_(1, 24 )_ = 4.335, *p* < 0.05; interaction *F*_(1, 24)_ = 10.25, *p* < 0.01). In the EPM test, the VPA-exposed offspring spent less time in the open arms, and ILPFC DBS reduced anxiety as compared with sham surgery in the VPA-exposed offspring (Figure 1C; saline/sham *n* = 7, saline/DBS *n* = 5 VPA/sham *n* = 9, VPA/DBS *n* = 7; two-way ANOVA, pre-DBS *F*_(1, 24)_ = 4.649, *p* < 0.05; post-DBS *F*_(1, 24)_ = 5.496, *p* < 0.05; interaction *F*_(1, 24)_ = 5.072, *p* < 0.05). In the open field test, in the VPA-exposed offspring under ILPFC DBS, hyperactivity was ameliorated relative to the control rats (Figure 1D; saline/sham *n* = 7, saline/DBS *n* = 5, VPA/sham *n* = 9, VPA/DBS *n* = 7; two-way ANOVA, pre-DBS *F*_(1, 24)_ = 7.659, *p* < 0.05; post-DBS *F*_(1, 24)_ = 13.34, *p* < 0.01; interaction *F*_(1, 24)_ = 15.36, *p* < 0.001).

### 2.2. Effect of DBS on Autistic Behaviors in VPA-Exposed Offspring Is Mediated by the Serotonin System

**Figure 2:**
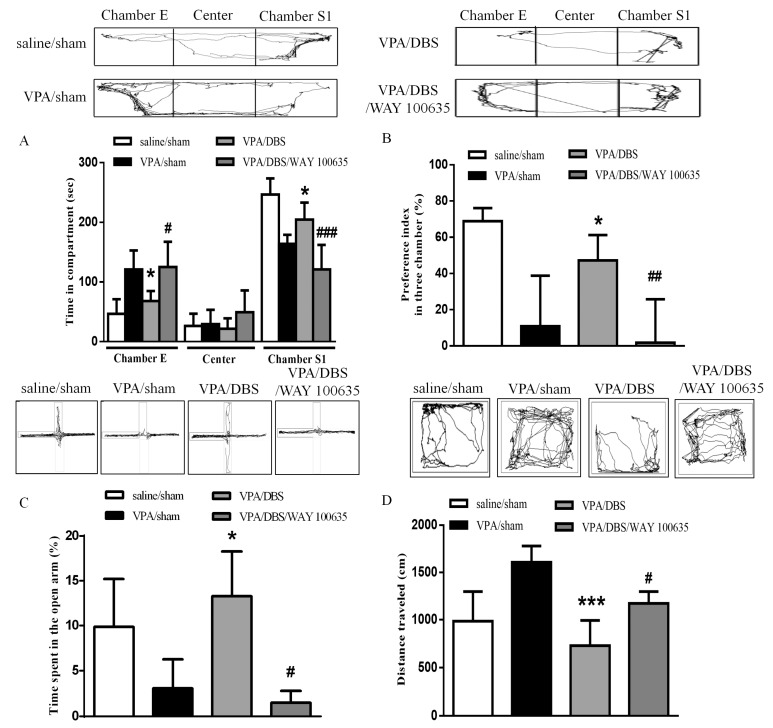
WAY 100635 blocked the improvements in social interaction, anxiety and hyperlocomotion instigated by ILPFCDBS in the VPA-exposed offspring. (**A**) Duration of latency on entering chamber E, the center chamber, and chamber S1 during the three-chamber social interaction test; (**B**) preference index in the social interaction test; (**C**) percentage of time spent in the open arms; (**D**) total distance traveled in the open field test. * *p* < 0.05 vs. VPA/sham; *** *p* < 0.05 vs. VPA/sham; ^#^ *p* < 0.05 vs. VPA/DBS; ^##^ *p* < 0.01 vs. VPA/DBS; ^###^ *p* < 0.001 vs. VPA/DBS. Sample sizes (*n*): saline/sham *n* = 7, VPA/sham *n* = 9, VPA/DBS *n* = 5, VPA/DBS/WAY 100635 *n* = 5.

In a previous study, chronic DBS engagement with an intact 5-HT system in the mPFC was reported; therefore, we tested the hypothesis by administering WAY 100635 (0.5 mg/kg i.p.) 30 min prior to ILPFC DBS [18]. The results showed that administration of WAY 100635 reduced both the time spent in the social zone and the social preference index after ILPFC DBS (time in chamber S1: 121.1 ± 20.46 s, *n* = 5; social preference index: 1.798 ± 9.794%, *n* = 5) as compared with the DBS group (time in chamber S1: 204.5 ± 11.69 s, *n* = 5, *p* < 0.001; social preference index: 47.22 ± 6.241%, *n* = 5, *p* < 0.01), suggesting that WAY 100635 blocked the reversing effects of DBS on sociability in the VPA-exposed offspring (Figure 2A,B). The increased time spent in the open arms after ILPFC DBS (13.27 ± 2.502%, *n* = 5, *p* < 0.05) was reversed by treatment with WAY 100635 in the VPA-exposed offspring (Figure 2C; 1.490 ± 0.755%, *n* = 5, *p* < 0.05). We found that the effect of ILPFC DBS (729.5 ± 79.49 cm, *n* = 5) in terms of reversing hyperlocomotor activity was eliminated by administration of WAY 100635 (Figure 2D; 1173 ± 62.30 cm, *n* = 5, *p* < 0.05).

### 2.3. Effects of 5-HT_1A_ Agonist 8-OH-DPAT on Autism-Related Behaviors in VPA-Exposed Offspring

**Figure 3:**
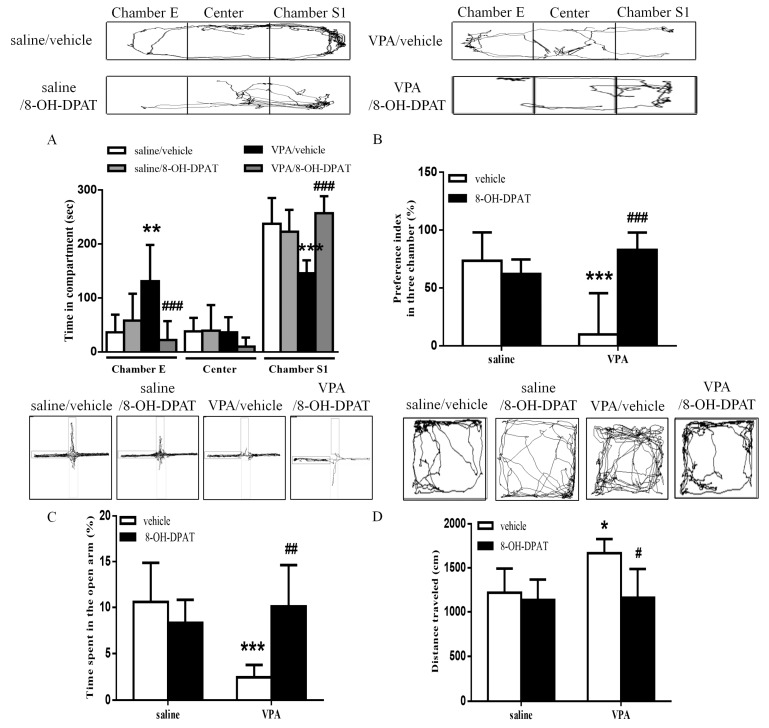
8-OH-DPAT treatment increased social interaction and ameliorated anxiety and hyperlocomotion in the VPA-exposed offspring. (**A**) Time spent by the VPA-exposed offspring in chamber E, the center chamber, and chamber S1 during the three-chamber social interaction test following local infusion of 8-OH-DPAT (10 μg/μL) into the ILPFC; (**B**) preference index in the social interaction test; (**C**) the percentage in the open arms after 8-OH-DPAT treatments; (**D**) bar chart revealing the total distance traveled in the open field test. * *p* < 0.05 vs. saline/vehicle; ** *p* < 0.01 vs. saline/vehicle; *** *p* < 0.001 vs. saline/vehicle; ^#^ *p* < 0.05 vs. VPA/vehicle; ^##^ *p* < 0.01 vs. VPA/vehicle; ^###^ *p* < 0.001 vs. VPA/vehicle. Sample sizes (*n*): saline/vehicle *n* = 6, saline/8-OH-DPAT *n* = 7, VPA/vehicle *n* = 8, VPA/8-OH-DPAT *n* = 5.

Next, we confirmed the involvement of serotonin in the regulation of abnormal behaviors. We administered the 5-HT_1A_ receptor agonist 8-OH-DPAT (10 μg/μL) into the ILPFC of the VPA-exposed offspring. The decreased social interaction with the stranger rat was improved after treatment with 8-OH-DPAT. Two-way ANOVA revealed pre-treatment (VPA vs. saline, *F*_(1, 22)_ = 12.30, *p* < 0.01), drug (8-OH-DPAT vs. vehicle, *F*_(1, 22)_ = 4.338, *p* < 0.05) and a pre-treatment by drug interaction (*F*_(1, 22)_ = 21.02, *p* < 0.001) (Figure 3A). We observed that 8-OH-DPAT improved the decreased social preference index in the VPA-exposed offspring (Figure 3B; saline/vehicle *n* = 6, saline/8-OH-DPAT *n* = 7, VPA/vehicle *n* = 8, VPA/8-OH-DPAT *n* = 5; two-way ANOVA, pre-treatment *F*_(1, 22)_ = 4.71, *p* < 0.05; drug *F*_(1, 22)_ = 9.786, *p* < 0.01; interaction *F*_(1, 22)_ = 18.29, *p* < 0.001). In the EPM test, 8-OH-DPAT treatment restored the decreased time spent in the open arms by the VPA-exposed offspring (Figure 3C; saline/vehicle *n* = 6, saline/8-OH-DPAT *n* = 7, VPA/vehicle *n* = 8, VPA/8-OH-DPAT *n* = 5; two-way ANOVA, pre-treatment *F*_(1, 22)_ = 6.267, *p* < 0.05; drug *F*_(1, 22)_ = 4.504, *p* < 0.05; interaction *F*_(1, 22)_ = 15.31, *p* < 0.001). In the open field test, two-way ANOVA revealed pre-treatment (VPA vs. saline, *F*_(1,22)_ = 5.644, *p* < 0.05), drug (8-OH-DPAT vs. vehicle, *F*_(1, 22)_ = 8.681, *p* < 0.01) and a significant pre-treatment by drug interaction (*F*_(1, 22)_ = 4.513, *p* < 0.05), suggesting that hyperlocomotor activity also improved after 8-OH-DPAT treatment (Figure 3D).

### 2.4. Decreased Duration of DBS Combined with 8-OH-DPAT Treatment Improved Sociability in VPA-Exposed Offspring

**Figure 4:**
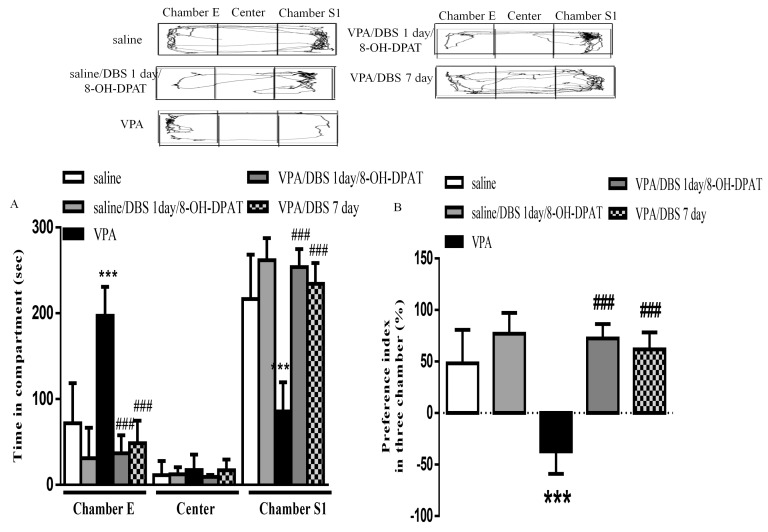
One-day ILPFC DBS combined with 8-OH-DPAT treatment facilitated improvement of social interaction and social preference in the VPA-exposed offspring. (**A**) Duration of latency on entering chamber E, the center chamber, and chamber S1 during the three-chamber social interaction test; (**B**) preference index in the social interaction test. *** *p* < 0.001 vs. saline-; ^###^ *p* < 0.001 vs. VPA. Sample sizes (*n*): saline *n* = 5, VPA *n* = 8, VPA/DBS 1 day/8-OH-DPAT *n* = 4, saline/DBS 7 days *n* = 5.

To confirm whether DBS improved autistic patterns via the serotonin system, we observed that one-day ILPFC DBS combined with 8-OH-DPAT treatment significantly reversed the impaired sociability; however, one-day ILPFC DBS had no effect on improving autistic behaviors (data not shown). We observed that when the duration of ILPFC DBS was reduced to one day, and was combined with 8-OH-DPAT treatment, the time spent in chamber S1 increased significantly (253.8 ± 10.58 s, *n* = 4, *p* < 0.001), similar to the effect of chronic DBS (234.3 ± 10.95 s, *n* = 5, *p* < 0.001), in the VPA-exposed offspring (Figure 4A). The results also showed that the impaired social preference index of the VPA-exposed offspring (-37.22 ± 7.73%, *n* = 8) was reversed following one day of ILPFC DBS combined with 8-OH-DPAT treatment (Figure 4B; 72.35 ± 7.033%, *n* = 4, *p* < 0.001).

### 2.5. DBS Restored the Excitatory and Inhibitory Balance via the 5-HT System

**Figure 5:**
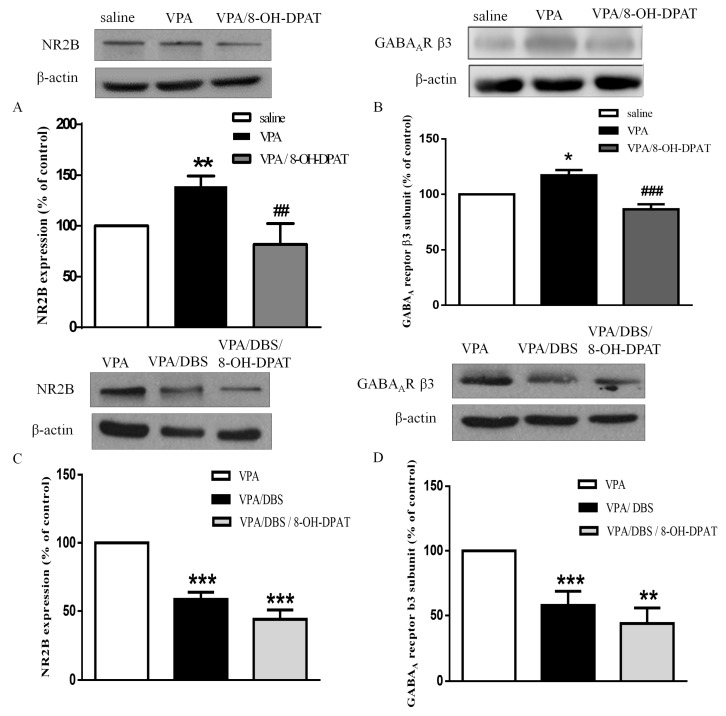
DBS and 8-OH-DPAT treatment reversed the increased expressions of NMDAR subunit NR2B and the GABA_A_R β3 subunit in the VPA-exposed offspring. (**A**) Representative blots and quantification showing the synapse NMDAR subunit NR2B expression after 8-OH-DPAT treatment in the VPA-exposed offspring. Sample sizes (*n*): Saline *n* = 12, VPA *n* = 9, VPA/8-OH-DPAT *n* = 5; (**B**) representative blots and quantification showing the synapse GABA_A_R β3 subunit expression after 8-OH-DPAT treatment in the VPA-exposed offspring. Sample sizes (*n*): Saline *n* = 4, VPA *n* = 4, VPA/8-OH-DPAT *n* = 4; (**C**) immunoblots and quantification showing the synapse NMDAR subunit NR2B expression after seven days of DBS and after three days of DBS combined with 8-OH-DPAT treatments in the VPA-exposed offspring. Sample sizes (*n*): VPA *n* = 14, VPA/DBS *n* = 12, VPA/DBS/8-OH-DPAT *n* = 12; (**D**) western blotting showing the synapse GABA_A_R β3 subunit expression after seven days of DBS and after three days of DBS combined with 8-OH-DPAT treatment in the VPA-exposed offspring. Sample sizes (*n*): VPA *n* = 8, VPA/DBS *n* = 6, VPA/DBS/8-OH-DPAT *n* = 6. * *p* < 0.05 vs. saline/vehicle; ** *p* < 0.01 vs. saline/vehicle; * *p* < 0.05 vs. saline; ** *p* < 0.01 vs. saline; ** *p* < 0.01 vs. VPA; *** *p* < 0.001 vs. VPA; ^##^ *p* < 0.01 vs. VPA; ^###^ *p* < 0.001 vs. VPA.

8-OH-DPAT administration reversed the enhancement of the levels of NMDAR subunit NR2B (81.79 ± 20.70% of saline, *n* = 5) and the GABA_A_R β3 subunit (86.58 ± 4.49% of saline, *n* = 4) in the VPA-exposed offspring (Figure 5A,B; NR2B: 139.0 ± 10.03% of saline, *n* = 9, *p* < 0.01; GABA_A_R β3: 117.2 ± 4.64% of saline, *n* = 4, *p* < 0.001). In addition, we found that DBS decreased the higher levels of NMDAR subunit NR2B (58.55 ± 5.27% of VPA, *n* = 11) and the GABA_A_R β3 subunit (59.97 ± 10.87% of VPA, *n* = 6) in the VPA-exposed offspring. Furthermore, three days of ILPFC DBS combined with 8-OH-DPAT treatment corrected the expressions of NMDAR subunit NR2B and the GABA_A_R β3 subunit, suggesting that DBS exerted similar signaling to 8-OH-DPAT to restore the E/I imbalance in the VPA-exposed offspring (Figure 5C,D; NR2B: 43.89 ± 6.97% of VPA, *n* = 11, *p* < 0.001; GABA_A_R β3: 43.95 ± 12.03% of VPA, *n* = 6, *p* < 0.01).

## 3. Discussion

Collectively, we have demonstrated that ILPFC DBS improves sociability, anxiety and hyperlocomotion via modulation of the 5-HT system in VPA-exposed offspring. The present study provided novel evidence of facilitation of social interaction by subsequent 8-OH-DPAT treatment following DBS for three days. Moreover, our findings underscore the functional importance of DBS orchestrating cognitive function via restoring the E/I imbalance by decreasing the expression of NMDAR subunit NR2B and the GABA_A_R β3 subunit in VPA-exposed offspring. 

In our previous study, we found that central thalamus- DBS increased cognitive function for lever-pressing skill learning via alteration of the striatal thalamic connectivity [42]. ILPFC DBS increased social interaction in olfactory bulbectomized (OBX) rats [43]. In addition, in both ASD mouse models *Viaat-Mecp2^−/y^* and *Shank3B^−/−^*, self-grooming was suppressed by DBS [44]. Socio-emotional deficits are known to be a core symptom of ASD. Clinical studies have reported subcallosal cingulate (SCC) DBS treatment for resistant depression [45,46]. SCC stimulation influences brain areas including the PFC, dorsal anterior cingulate and thalamus, which regulate emotional processing for treatment in an animal model of depression [47]. In addition, SCC stimulation increased social interaction and reduced negative emotional processing [48]. Previous study revealed that PFC DBS had antidepressant-like effects, and in our current animal study it was observed that PFC DBS improved sociability [21]. Thus, PFC stimulation may activate the brain areas of the SCC that are involved in regulating cognitive function and emotional behaviors in the VPA-exposed offspring. DBS has already been used for the treatment of neurological disorders including PD, hyperkinetic disorders and epilepsy [49,50]. Previous studies demonstrated that subthalamic nucleus (STN) DBS increased striatal dopamine metabolites and alleviated motor function [51,52]. We observed that ILPFC DBS rescued the hyperlocomotor activity in the VPA-exposed offspring. However, the effect of STN-DBS in terms of dopamine, and whether or not this influences motor or other behaviors in VPA-exposed offspring still needs to be investigated in further study.

Previous studies have observed that ASD patients exhibit enhanced blood serotonin levels due to alteration of production and uptake function by enterochromaffin cells and platelets, respectively [53]. Muller et al. suggested that the increased serotonin storage and uptake in the presynapse results in decreasing synaptic serotonin [29]. N-acetylserotonin (NAS), an intermediate of the serotonin metabolism and a precursor in melatonin synthesis, has been reported to be increased in ASD. Furthermore, the concentration of serotonin-derived metabolite melatonin is reduced in ASD patients as compared with controls [54,55]. Previous study observed hyperserotonemia, in addition to increased NAS and a deficit in melatonin, suggesting impairment of the serotonin-NAS-melatonin pathway as a biomarker of ASD [56]. However, whether alterations of NAS or melatonin expression exist in the PFC of VPA-exposed offspring requires further investigation. Previous study revealed that systemic administration of 5-HT_1A_ receptor agonist 8-OH-DPAT reduced anxiety, as per the social interaction paradigm [57,58]. Furthermore, 8-OH-DPAT also selectively activated 5-HT_7_ receptors, which has been reported to improve metabotropic glutamate receptor-dependent synaptic plasticity in Fragile X syndrome [59]. In this study, we found that administration of 8-OH-DPAT improved autistic behaviors, with similar effects to those of DBS treatment. 8-OH-DPAT acts on both 5-HT_1A_ and 5-HT_7_ receptors; hence, we could not rule out the possibility that 5-HT_7_ receptors were involved in this study. Although the effect of DBS treatment was blocked by application of a selective 5-HT_1A_ receptor antagonist that was more selective for 5-HT_1A_ receptors than other 5-HT subtypes, we still cannot exclude the involvement of other 5-HT subtypes in this study. The detailed mechanism of the action of 8-OH-DPAT on the 5-HT_1A_ receptors or 5-HT_7_ receptors in the VPA-exposed offspring merits further investigation. Previous studies have reported that PD is often accompanied by depressive disorder [60,61]. Hameleers first demonstrated that the effect of STN-DBS is mediated by a 5-HT-dependent mechanism, which treats PD-related depression by inhibiting 5-HT release [62,63,64]. Previous study revealed that activation of ILPFC DBS elevated 5-HT in the mPFC, accompanied by an antidepressant effect [43,65]. Furthermore, DBS restored the social approach by regulating the E/I synaptic inputs onto 5-HT neurons [21]. In our study, we found that DBS improved autistic behaviors that were disrupted by a 5-HT_1A_ receptor antagonist, suggesting that the effect of DBS occurred by regulating the serotonin system in the VPA-exposed offspring. Our data supported effects of DBS via restoring the serotonin system; acute DBS for three days with subsequent 8-OH-DPAT administration facilitated social behavior improvement in the VPA-exposed offspring. In accordance with these results, another study demonstrated that the effect of DBS in terms of an anticonvulsant effect was facilitated by blocking of serotonin antagonist METH, supporting the idea that DBS alters the serotonin-mediated synaptic function [66]. In our study, we observed that one-day DBS combined with 8-OH-DPAT treatment significantly improved the impaired sociability; however, we were unable to ascertain whether the effects of 7 days of DBS combined with 8-OH-DPAT treatment would enhance, worsen or leave unchanged the DBS expression. Clinical study showed that globus pallidus interna (GPi) stimulation combined with levodopa treatment improved PD symptoms for 5 years. In contrast, the improvement of PD symptoms with STN stimulation combined with levodopa treatment decreased [67]. The differences in results obtained when DBS is combined with medications may due to GPi having a direct connection to the motor cortex via the thalamus [68]. Although PFC DBS is used to treat ASD, we cannot exclude other brain areas being more superior sites for the treatment of cognitive function deficits in VPA-exposed offspring. In addition, we cannot rule out the possibility that PFC DBS for 7 days combined with 8-OH-DPAT treatment may activate other socio-emotional networks including the limbic system, facial processing or the mirror neuron network [11]. In our study, we found that combining medication and DBS decreased the number of days of treatment required, which is very important in terms of decreasing the long-term side effects of medication or DBS in the clinical setting. However, the effect of 7 days of combined DBS and 5-HT1_A_ receptor agonist treatment could provide a direction of treatment in VPA-exposed offspring that merits further investigation.

Collectively, the available data suggest that the serotonin system is an important system that is modulated by DBS to improve autistic behaviors in VPA-exposed offspring. Dysfunction of NMDAR expression and GABAergic synaptic transmission have been reported in VPA-induced offspring at an early developmental age [69,70]. Similarly, our results showed that DBS modulation decreased the expressions of NMDAR subunit NR2B and the GABA_A_R β3 subunit in the PFC of VPA-exposed offspring. Previous study reported decreased expressions of NMDAR and GABAergic proteins in VPA-induced autistic adolescent mice; however, this may involve a compensatory mechanism resulting in hypo-functioning synapse [71]. A previous study demonstrated that high-frequency DBS induced dyskinesia by activating NMDAR subunit NR2B, while another study revealed that DBS with stimulation in the frequency range of 100–200 Hz maximally activated extrasynaptic NMDARs subunit NR2B [72,73]. Previous studies revealed that DBS not only reduced the concentration of glutamate, but also the expression of GABA, during cognitive testing [74,75]. High-frequency stimulation suppresses spontaneous neuronal firing, suggesting that the stimulus intensity may regulate the inhibitory interneurons [76]. Hence, we cannot rule out the parameter of stimulus intensity whether superior for modulation of the E/I in ASD. The serotonin system modulates neurotransmitter release, including glutamate and GABA depending on the distinct serotonin receptor subtypes [77]. In addition, the serotonin system regulates GABA inhibition in the PFC which is considered to modulate cognitive function in ASD [3,28]. VPA-exposed offspring reveal abnormal serotonergic neuronal differentiation and migration [38]. Impairment of GABAR and NMDAR synaptic expression leads to E/I imbalance in VPA-exposed offspring [70,78]. Hence, we speculated that DBS modulates dysfunction of the serotonin system, which may correct the E/I imbalance. In addition, 5-HT_1A_ receptors are known to depress GABA and glutamate synaptic signaling in the nucleus tractus solitarii [79]. Activation of 5-HT_1A_ receptors reduces the surface NR2B level via the mechanism of microtubule dynamics, which is regulated by Ca^2+^ /calmodulin-dependent protein kinase II (CaMKII) and the extracellular signal-regulated kinase (ERK) signaling pathway [80]. Our results demonstrated that DBS combined with 8-OH-DPAT treatment also decreased the expressions of NMDAR subunit NR2B and the GABA_A_R β3 subunit, suggesting that ILPFC DBS has a similar signaling pattern, activating 5-HT_1A_ receptors in VPA-exposed offspring. However, whether alteration of the microtubule-dependent mechanism in the PFC of VPA-exposed offspring occurs after DBS treatment requires further study. Taken together, we have provided evidence to show that DBS improves autistic behaviors by modulating the E/I balance via 5-HT_1A_ receptors. Thus, modulation of the serotonergic system by DBS may be a potential strategy by which to improve ASD.

## 4. Materials and Methods

### 4.1. Animals

Pregnant Sprague Dawley rats received an injection of valproic acid (500 mg/kg) on gestational day 12.5 as previously described [81]. The offspring were housed four to five in a temperature-controlled (25 °C) cage under a light–dark cycle with food and water available *ad libitum.* 4–5-weeks-old of male saline- and VPA-exposed offspring were used in the experiments. All procedures were approved by the Experimental Animal Review Committee at National Yang-Ming University on 1 July 2017 (1060433r, for project “The mechanism and treatment on valproic acid induced the imbalance of excitatory/inhibitory in the autism animal model).

### 4.2. DBS Surgeries and Experiments

**Figure 6:**
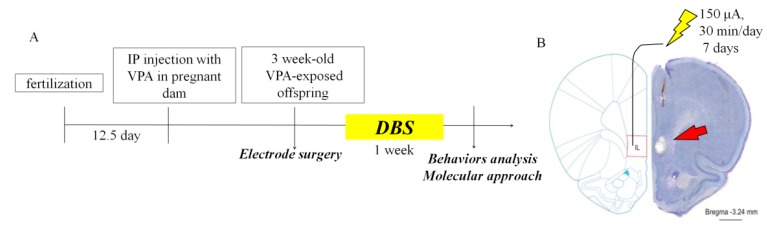
Experimental design of ILPFC DBS applied to the VPA-exposed offspring. (**A**) Schematic representation flow chart of the experimental design; (**B**) verification of unilateral DBS electrode placement on the right-hand side by Nissl staining of the ILPFC. Scale bar, 1 mm.

Male VPA-exposed offspring were operated on under anesthesia, with ketamine (100 mg/kg, i.p.) administered during surgery. A multi-array, stainless steel wire electrode (California Fine Wire Company, Grover Beach, CA, USA) was implanted unilaterally into the right mPFC (2.2 mm anteroposterior, 0.5 mm mediolateral, 4.8 mm dorsoventral), which has been reported to be effective for bilateral stimulation, and unilateral stimulation, and also reduces morbidity during surgery [82]. We applied cyanoacrylate and dental cement to affix the electrodes, as previously described [83]. After a one–week period of recovery from surgery, the implanted electrodes were connected to a programmable stimulator (A-M system, Sequim, WA, USA). Stimulation was applied at a frequency of 160 Hz with a 60-μs pulse width and 150-μA current. DBS was applied for 30 min per day for 7 days while rats remained in their home cages. After 7 days of DBS, rats underwent social interaction testing 24 h after the end of stimulation. Shams had electrodes implanted and retained in the mPFC but did not receive stimulation. The animals were left undisturbed in their home cages for 7 days after the surgery, except for normal handling for cage cleaning. Figure 6A depicts the experimental design used to study the effects of DBS on behavior in VPA-exposed offspring.

### 4.3. Chemicals

8-OH-DPAT (0.5 mg/kg i.p.) was obtained from SIGMA-Aldrich and WAY 100635 (0.5 mg/kg i.p.) from Tocris Bioscience (Bristol, UK).

### 4.4. Behavioral Testing

All behavioral trace of rat movements during each experiment were recorded using smart software (version 3.0; Panlab, S.L.U. Barcelona, Spain).

### 4.5. Three-Chamber Social Interaction Test

The three-chamber social interaction test was adapted from Crawley [84] and performed using 4-week-old saline- and VPA-exposed rat offspring as we reported previously [81]. The apparatus had three compartments: the right compartment, where a stranger rat was placed under a plastic box (27 × 13 × 20 cm designated “chamber S1”); the left compartment, in which had nothing had been placed in the plastic box (designated “chamber E”); and the central compartment. After habituation in the testing room, the test rats were placed into the central compartment for 5 min. The preference score was defined as the time spent with stimulus subtracted from the time spent without stimulus, divided by the whole duration of the test.

### 4.6. Elevated Plus Maze (EPM)

The EPM consisted of a plus-shaped apparatus (112 × 112 × 31 cm with two open arms and two closed arms. The percentage of time spent in the open arms was recorded over a 10 min period.

### 4.7. Open Field Test

The offspring were placed in a black plastic box (45 × 45 × 45 cm) and the total distance travelled was measure for 5 min.

### 4.8. Nissl Staining

Rats were deeply anaesthetized with ketamine (10 mg/100 g) and transcranially perfused with 4% paraformaldehyde (PFA). Vibratome brain sections (50 μm) were obtained and dried on slides overnight. Sections were immersed in 1:1 alcohol/chloroform for 8 h and then rehydrated through 100% to 95% alcohol to distilled water (10 min each step). Sections were stained in 0.1% cresyl violet at 37 °C for 10 min and rinsed in distilled water. The sections were then dehydrated and mounted on slides using a mounting medium (Fluoromount-G, Southern Biotech, Birmingham, AL, USA), and images were observed using a fluorescence microscope (BX63; Olympus, Tokyo, Japan). To verify the correct positioning of DBS in the rats, we performed Nissl staining of the implanted electrodes in the IL area (Figure 6B).

### 4.9. Western Blotting Analysis

Brain tissues were lysed in a lysis buffer (1% Triton X-100, 0.1% SDS, 50 mM Tris-HCl, pH 7.5, 0.3 M sucrose, 5 mM EDTA, 2 mM sodium pyrophosphate, 1 mM sodium orthovanadate, and 1 mM enylmethylsulfonyl fluoride) containing a complete protease inhibitor cocktail. The samples were centrifuged at 12,000 rpm for 30 min to obtain supernatants. The supernatants were assayed using a Bradford assay kit then separated by SDS-PAGE electrophoresis and transferred to Immobilon-P membranes (Millipore, Billerica, MA, USA). The membranes were incubated in 5% nonfat dry milk for 60 min, then incubated with anti-beta actin antibody (1:10,000, Abcam, Cambridge, UK), anti-NR2B antibody (1:2000; Millipore, Burlington, MA, USA) and anti-GABA_A_R β_3_ antibody (1:5000; Abcam, Cambridge, UK) overnight at 4 C. The membranes were then incubated with HRP-conjugated secondary antibodies for 1 h at room temperature followed by treatment with ECL Plus detection reagent (PerkinElmer, Boston, MA, USA). Protein levels were first normalized to the internal control level for each sample.

### 4.10. Statistical Analysis

All data are expressed as the mean ± SEM. Significances of differences between groups were calculated using one-way analysis of variance (ANOVA) or two-way ANOVA, followed by Bonferroni *post hoc* comparison testing. Probability values (*p*) of less than 0.05 were considered to represent significant differences.
